# Data on factors influencing the cost, time performance of the Industrialized Building System

**DOI:** 10.1016/j.dib.2018.04.036

**Published:** 2018-04-18

**Authors:** Ayodeji O. Ogunde, Rachael Ayodele, Opeyemi Joshua, David O. Nduka, Abisola Ogunde, Kunle E. Ogundipe, Babatunde F. Ogunbayo, Adekunle M. Ajao

**Affiliations:** a Department of Building Technology, Covenant University, Ota, Ogun State, Nigeria; b Department of Estate Management, Covenant University, Ota, Ogun State, Nigeria

## Abstract

The data article provides the factors that influence the cost; time performance of the Industrialized Building System (IBS), its prospects and challenges. A survey technique was used for this research. Structured Questionnaires were administered to occupants of prefabricated buildings and interviews were conducted with the professionals in the building industry. Statistical Package for Social Sciences (SPSS version20) was used to analyse the data obtained from the questionnaires. The variables were ranked based on Relative Importance Index (RII) calculation. The Data indicated that IBS would be more economical if used for mass production.

**Specifications Table**

**Table:** 

Subject area	*Building Construction*
More specific subject area	*Construction Management*
Type of data	*Table, Text file, figure*
How data was acquired	*Field survey*
Data format	*Raw, filtered, analyzed data*
Experimental factors	*Statistical Package for Social Sciences (SPSS version20) was used for this research. Statistical tool such as descriptive tool was used to analyse the data obtained from questionnaires. Relative Importance Index (RII) was used to rank the variables.*
Experimental features	*Data was obtained from structured questionnaires administered to occupants of prefabricated buildings and interviews were conducted with the professionals in the building industry. Secondary data was also obtained from published journals, articles and thesis related to the subject of this research. The data was collated and analysed, using mean item score ranking, percentages and descriptive statistics.*
Data source location	*Lagos State, Nigeria.*
Data accessibility	*Data is available with this article*
Related research article	*Ogunde A.O; Selekere T. E; Joshua, O; Kukoyi, P. O; Omuh, I.O Prefabrication Method of Building Construction in Lagos State, Nigeria: Prospects and Challenges, International Journal of Engineering Technology and Computer Research (IJETCR), Volume 4; Issue 1; January-February-2016; Page No. 88–100, Available Online at*www.ijetcr.org*[1]*

**Value of the data**

• This data provides a benchmark for further studies on the performance of the precast concrete system in the construction industry.
• This data shows the significance of using Industrial Building Systems (IBS), its impact and valuable benefits of incorporating it in the industry.
• This data shows cost- time effectiveness and perception of the implementation of the building system for policy making.
• This data can be used to compare findings from other countries where this type of construction method is prevalent.

## 1 Data

The data article provides the factors that influence the cost; time performance of the Industrialized Building System (IBS), its prospects and challenges.

## 2 Experimental design, materials and methods

### 2.1 Data collection

Data was obtained from building professionals involved in the construction of precast concrete structures and 60 questionnaires were distributed to occupants of already constructed precast buildings in Lagos state. Data was also collected from secondary sources which include information from published journals, articles and thesis [1], [2], [3], [4], [5], [6], [7], [8], [9], [10], [11], [12].

### 2.2 Data analysis and presentation

Data collected from the questionnaires administered to respondents were analysed and presented as follows. Table 1 shows the response rate, Table 2 shows the gender of the occupants, Table 3 shows years of occupying the building, Table 4 shows status of ownership, Table 5 shows monthly income of occupants. Table 6 is on awareness of method of construction, Table 7 shows sources of information, Table 8 is on perception of occupants on cost performance, Table 9 is on susceptibility of building type to collapse and defects, while Table 10 shows the satisfaction of occupant, finally, Table 11 shows the challenges encountered by building occupants. Other relevant literature are can be found in [13, [14], [15], [16], [17], [18], [19], [20], [21], [22], [23], [24].

**Table 1:** Response rate of occupants of prefabricated buildings.

S/N	Questionnaires	Prefab building occupants
1	Administered	85
2	Returned	60
3	Response rate	70%

**Table 2:** Gender of occupants of prefabricated buildings.

S/N		Frequency	Percent
1	Male	45	75.0
2	Female	15	25.0
	Total	60	100.0

**Table 3:** Years of occupying the building by respondents.

S/N	Years	Frequency	Percent
1	1–5 years	18	30.0
2	6–10 years	20	33.3
3	11–15 years	15	25.0
4	16–20 years	4	6.7
	Total	57	95.0
Missing	System	3	5.0
Total		60	100.0

**Table 4:** Status of ownership of prefabricated buildings.

S/N	Ownership	Frequency	Percent
1	Owned	31	51.7
2	Rented	29	48.3
	Total	60	100.0

**Table 5:** Monthly incomes of occupants of IBS buildings.

S/N	Income range	Frequency	Percent
1	Below 500,000	24	40.0
2	500,000–700,000	10	16.7
3	700,000–900,000	17	28.3
4	above 900,000	3	5.0
	Total	54	90.0
Missing	System	6	10.0
Total		60	100.0

**Table 6:** Awareness of method of construction of IBS.

S/N	Awareness	Frequency	Percent
1	Yes	40	66.7
2	No	20	33.3
	Total	60	100.0

**Table 7:** sources of information on IBS.

S/N	Sources	Frequency	Percent
1	Formal education	22	36.7
2	Personal study	4	6.7
3	Construction personnel	7	11.7
4	Others	7	11.7
	Total	40	66.7
Missing	System	20	33.3
Total		60	100.0

**Table 8:** Perception of occupants on cost performance of IBS.

S/N	Cost perception	Frequency	Percent
1	Less expensive	8	13.3
2	Expensive	23	38.3
3	Moderately expensive	18	30.0
4	Highly expensive	11	18.3
	Total	60	100.0

**Table 9:** Susceptibility of building type to collapse and defects.

S/N		Frequency	Percent
1	Yes	5	8.3
2	No	55	91.7
	Total	60	100.0

**Table 10:** Satisfaction of occupants of prefabricated buildings.

S/N	Satisfaction	Frequency	Percent
1	Not satisfied	4	6.7
2	Satisfied	16	26.7
3	Moderately satisfied	29	48.3
4	Highly satisfied	9	15.0
5	Neutral	2	3.3
	Total	60	100.0

**Table 11:** Challenges encountered by building occupants of IBS buildings.

S/N	Challenges encountered in the use of this method	Mean	RII	Ranking
1	Inability to make changes to building after it has been installed	3.97	0.794	1
2	Leakages from joints of building element	3.95	0.79	2
3	Initial high cost of financing projects using this method	3.48	0.696	3
4	Services e.g. electrical and plumbing challenges	3.27	0.654	4
5	Inadequate ventilation system	3.23	0.646	5
6	Limited availability of personnel to repair damages	3	0.6	6
7	Expensive to maintain or repair	2.92	0.584	7
8	Problem of cracks and dampness on wall	2.68	0.536	8
9	Poor sound control	2.47	0.494	9
